# Improving the Barrier Properties of Packaging Paper by Polyvinyl Alcohol Based Polymer Coating—Effect of the Base Paper and Nanoclay

**DOI:** 10.3390/polym13081334

**Published:** 2021-04-19

**Authors:** Zhenghui Shen, Araz Rajabi-Abhari, Kyudeok Oh, Guihua Yang, Hye Jung Youn, Hak Lae Lee

**Affiliations:** 1 Program in Environmental Materials Science, Department of Agriculture, Forestry and Bioresources, College of Agriculture and Life Sciences, Seoul National University, Seoul 08826, Korea; zhshen@snu.ac.kr (Z.S.); araz61@snu.ac.kr (A.R.-A.); page94@snu.ac.kr (H.J.Y.); 2 Research Institute of Agriculture and Life Sciences, Seoul National University, Seoul 08826, Korea; ogd0310@snu.ac.kr; 3 State Key Laboratory of Biobased Material and Green Papermaking, Qilu University of Technology, Shandong Academy of Sciences, Jinan 250353, China; ygh@qlu.edu.cn

**Keywords:** packaging paper, barrier properties, polyvinyl alcohol, alkyl ketene dimer, nanoclay

## Abstract

The poor barrier properties and hygroscopic nature of cellulosic paper impede the wide application of cellulosic paper as a packaging material. Herein, a polyvinyl alcohol (PVA)-based polymer coating was used to improve the barrier performance of paper through its good ability to form a film. Alkyl ketene dimer (AKD) was used to enhance the water resistance. The effect of the absorptive characteristics of the base paper on the barrier properties was explored, and it was shown that surface-sized base paper provides a better barrier performance than unsized base paper. Nanoclay (Cloisite Na^+^) was used in the coating formulation to further enhance the barrier performance. The results show that the coating of PVA/AKD/nanoclay dispersion noticeably improved the barrier performance of the paper. The water vapor transmission rate of the base paper was 533 g/m^2^·day, and it decreased sharply to 1.3 g/m^2^·day after the application of a double coating because of the complete coverage of the base paper by the PVA-based polymer coating. The coated paper had excellent water resistance owing to its high water contact angle of around 100°. The grease resistance and mechanical properties of the base paper also improved after coating. This work may provide inspiration for improving the barrier properties of packaging paper through the selection of a suitable base paper and coating formulation.

## 1. Introduction

Packaging materials play important roles in many industries, such as the food industry and pharmaceutical industry, as well as in people’s daily life. The basic function of packaging materials is to protect the contents from contaminants, leakage and damage in the production, storage, transportation and selling processes, and to provide safety, convenience and economic benefits [1]. Plastic, glass, metal and paper are the most commonly used packaging materials, among which plastic has been predominant in the global packaging market. Although plastic materials are light, flexible and strong, most of these petroleum-based products are nonrenewable and nonbiodegradable and have caused serious environmental issues [2]. A huge amount of plastic waste, including plastic packaging, is polluting the land and oceans and endangering ecosystems and the wildlife they contain. Paper packaging materials made from cellulosic fibers have the advantages of abundant availability, biodegradability, renewability and recyclability [3]. Furthermore, paper packaging materials are flexible, low cost and safe [4], and their good printability and ease of functionalization make them competitive in the packaging field. With rising awareness of environmental protection and sustainable development in society, paper-based packaging is attracting ever-increasing interest.

The barrier properties of packaging materials are critical for the protection of some products, such as moisture-sensitive foods and electronic devices. In this respect, cellulosic paper is not a perfect material for packaging owing to its poor barrier properties, which can mainly be ascribed to its porous three-dimensional structure. Because of the pores present both on the surface and inside the paper, molecules can diffuse through paper packaging rather easily and spoil the contents inside. Therefore, the porous nature prevents paper from fulfilling the protective function of a packaging material, thus limiting its application in the packaging sector. Additionally, the poor water resistance of paper, originating from the hydroxyl groups of cellulosic fibers, largely constrains its use as a packaging material in humid conditions. Upon contact with water, hygroscopic paper deteriorates and loses strength due to water absorption [5]. 

Intensive efforts have been made to improve the barrier properties of paper and paperboard products. Lavoine et al. [6] studied the effect of microfibrillated cellulose (MFC) on the barrier properties of paper and found that the air resistance of paper improved noticeably after a coating of MFC was applied. However, the grease resistance of the MFC-coated paper was not satisfactory. Mousavi et al. [7] coated cellulose nanofiber and carboxymethyl cellulose blends on paperboards and concluded that the coating improved the structure and barrier properties of the paper considerably. This coating also greatly improved the air resistance of paper, but the water vapor transmission rate (WVTR) and grease resistance of coated papers remained unsatisfactory. Hult et al. [8] attempted to enhance the barrier properties of paperboard by applying a lignin-based coating and found that the WVTR of the paperboard decreased from 840 to 240 g/m^2^∙day after the coating. Although improvements to the barrier properties of paper or paperboard through surface coating have been made in several studies, it is necessary to establish a better approach for coating in order to use paper products for the packaging of moisture-sensitive substances. In addition, the water resistance of paper, which is an important property for packaging applications, has been neglected in many studies. Hence, the fabrication of packaging paper with excellent barrier properties and water resistance remains an important objective.

To improve the barrier performance of porous paper, one effective method is to seal the pores on the paper’s surfaces and thereby block the diffusion pathway of liquid/gas molecules through the paper. A common protocol is to coat polymers with good film-forming performance on the paper substrate and thus form a continuous and impermeable film. Polyvinyl alcohol (PVA) is widely used for its various advantages, including excellent film-forming ability and good barrier performance and mechanical properties. The film-forming ability of PVA-based coatings on paper surfaces improves the barrier performance because the continuous PVA film blocks the pores on the surface and hinders the diffusion of permeating molecules. However, a PVA coating tends to have poor water resistance owing to the hydrophilic and water-soluble nature of PVA. In this study, alkyl ketene dimer (AKD), which has been extensively used as a hydrophobizing agent in paper mills to enhance the water resistance of paper, was added to the PVA coating to improve the hydrophobicity of PVA-coated papers. The combined use of PVA and AKD to improve the barrier performance of paper was tested in our previous work [9]. Our results showed that the WVTR of paper decreased considerably, from 543 to 2 g/m^2^∙day, when we applied a PVA/AKD triple coating. However, the PVA/AKD coating has a disadvantage in terms of the cost-effectiveness of decreasing the WVTR. The addition of a nanofiller (e.g., nanoclay) has been considered an efficient way to enhance the barrier property of polymer-based coating materials. Several studies [10,11,12] have demonstrated the effectiveness of nanoclay in improving barrier performance through the tortuosity effect. 

In the present study, PVA-compatible nanoclay (Cloisite Na^+^) was used as a nanofiller of a PVA-based polymer coating. By applying the PVA/AKD/nanoclay ternary coating material on the surface of paper, we aimed to prepare packaging paper with notably improved barrier performance and water resistance. Before coating the paper with this ternary dispersion, the influence of the base paper on improving the barrier performance was explored. Then, the effect of the PVA/AKD/nanoclay coating on the barrier performance of the paper was investigated to find cost-effective ways of fabricating packaging paper with superior barrier and water resistance properties. 

## 2. Materials and Methods

### 2.1. Materials

Two types of base paper made of bleached eucalyptus kraft pulp with and without surface sizing were kindly provided by Moorim Paper Co. Ltd. (Jinju, Korea). The surface sizing was performed by applying 3 g/m^2^ of oxidized starch to the surface of the dried web using a size press. The basic properties of these base papers are listed in Table 1. PVA (Polinol F17A, molecular weight = 75,000–80,000, degree of hydrolysis = 98–99.5%) was manufactured by OCI Company Ltd. (Seoul, Korea). AKD emulsion (HerconTM WI sizing agent) with a milky appearance and a solids content of 19.62% was supplied by Solenis Korea Ltd. (Gimcheon, Korea). Acridine orange (AO) was purchased from Samchun Chemical Co., Ltd. (Seoul, Korea). Nanoclay (Cloisite Na^+^) was obtained from Southern Clay Products Inc. (Gonzales, TX, USA). EpoFix resin (Struers, Cleveland, OH, USA) was used for embedding the coated samples. All of the materials and chemicals were used as received without any further purification or modification.

### 2.2. Methods

#### 2.2.1. Preparation of Coating Dispersions

The coating formulations are shown in Table 2. A solution with 8% PVA was prepared by dissolving the required amount of PVA powder in deionized water at 90 °C for 1 h, during which the dispersion was mixed with a mechanical stirrer (WiseStir, Daihan Scientific Co., Ltd., Wonju, Korea) at a speed of 800 rpm. To the PVA solution, the AKD emulsion was added and the mixture was mechanically mixed at 900 rpm for 1 h. Then, nanoclay was added and sonication with a frequency of 28 kHZ was conducted for 2 h using an ultrasound cleaner (SH-2300 Model, Saehan Ultrasonic, Seoul, Korea). Three levels of nanoclay dosage (i.e., 3%, 5% and 10%) were applied in the coating formulations. The prepared PVA/AKD/nanoclay dispersions were mechanically mixed at 1000 rpm at room temperature for 1 h before coating.

#### 2.2.2. Coating Process

The coating was carried out with an autobar coater (GBC-A4, GIST Co., Daejeon, Korea). The coating speed was 70 mm/s. Coated papers were dried in a hot air dryer (HB-502P0, Hanbaek Scientific Co., Bucheon, Korea) for 3 min and conditioned following TAPPI standard T 402 sp-13 (TAPPI 2013). The properties of the coated papers were then determined.

#### 2.2.3. Confocal Laser Scanning Microscope (CLSM) Imaging

The penetration of the coating materials into the base papers was investigated using a CLSM (SP8 X, Leica, Wetzlar, Germany). To this end, PVA was stained with AO in darkness for 6 h and then coated on the surfaces of the base papers in darkness. The coated papers were embedded in epoxy resin, followed by the curing (room temperature) and polishing (LaboPo-5 polisher, Struers, Cleveland, OH, USA) of the prepared resin blocks. The cross-sections of coated papers were examined under the CLSM and the acquired CLSM images were processed using software Leica Application Suite X (LAS X, Version 3.5.1, Leica, Wetzlar, Germany).

#### 2.2.4. Surface Morphologies of Papers

The surface morphologies of the papers were recorded by field-emission scanning electron microscopy (FE-SEM, Supra 55VP, Carl Zeiss, Oberkochen, Germany) with an acceleration voltage of 2 kV. The platinum coating of samples was performed before FE-SEM imaging using a sputter coater (EM ACE200, Leica, Vienna, Austria).

#### 2.2.5. Fourier-Transform Infrared (FT-IR) Spectroscopy

FT-IR spectra were recorded using a Nicolet 6700 spectrometer equipped with an attenuated total reflection accessory (Thermo Scientific, Waltham, MA, USA). The spectra were acquired with 32 scans in the range of 4000–1000 cm^−1^ at a resolution of 8 cm^−1^.

#### 2.2.6. Measurements of the Water Contact Angle (WCA)

The WCAs of the papers were determined using a drop shape analyzer (DSA100, Krüss GmbH, Hamburg, Germany). WCAs were measured for 60 s and the measurement interval was 1 s. Deionized water was used as the testing liquid and the volume of the water droplets was 5 µL. Six measurements were carried out for each sample and the average values were then calculated and reported.

#### 2.2.7. Measurements of the Cobb60 Value 

The Cobb60 values of the papers were measured at 23 °C and 50% relative humidity (RH) according to TAPPI standard T 441 om-13 (TAPPI 2013). The average of at least three measurements was calculated.

#### 2.2.8. Measurements of the WVTR and Grease Resistance

The WVTRs of the papers were measured at 23 °C and 50% RH following TAPPI standard T 448 om-17 (TAPPI 2017). Grease resistance measurements of the papers were performed according to TAPPI standard T 559 cm-12 (TAPPI 2012). At least three measurements were made for each paper and the average values were reported. 

#### 2.2.9. Mechanical Properties of Papers

The tensile properties of the papers were examined using a universal testing machine (Instron 5943, Instron Corp., Norwood, MA, USA) equipped with a 500-N load cell. Specimens were 15 mm in width and 100 mm in length and the tensile testing was carried out at 23 °C and 50% RH. Average values of the elongation at break and tensile strength were calculated and reported.

## 3. Results and Discussion

### 3.1. Comparison of the Properties of the Two Base Papers

**Figure 1:**
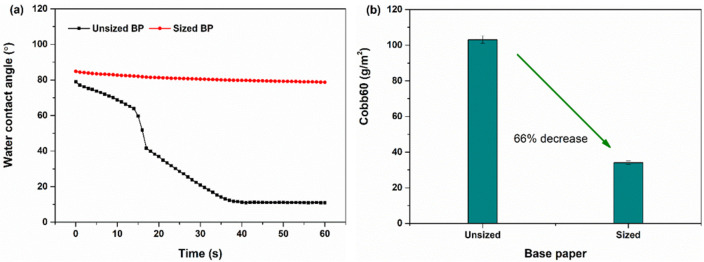
Characteristics of the two base papers: (**a**) WCAs and (**b**) Cobb60 values. BP = base paper.

The two base papers were similar in terms of several properties (Table 1), including the basis weight, thickness, bulk, density, roughness, brightness and WVTR. However, the liquid absorptive characteristics (Figure 1) differed greatly. The WCA results (Figure 1a) show that the two base papers had similar WCAs at the initial moment of testing. As time advanced, the WCA of the surface-sized base paper largely remained constant. In contrast, the WCA of the unsized base paper decreased sharply with testing time, finally reaching 11°, which is a value much lower than the WCA of the surface-sized base paper. The unsized base paper had poor water resistance due to the hydrophilic and porous nature of paper [13], which will allow easier absorption and penetration of water-borne coatings into the paper. The result of Cobb60 testing (Figure 1b) confirmed the different liquid absorptive performances of the two base papers. This difference may lead to different absorption or penetration of coating materials into the different base papers, which would consequently affect the barrier performance of PVA-based coated papers even for the same coat weight.

### 3.2. Effect of the Base Paper on the WVTR of Coated Papers

**Figure 2:**
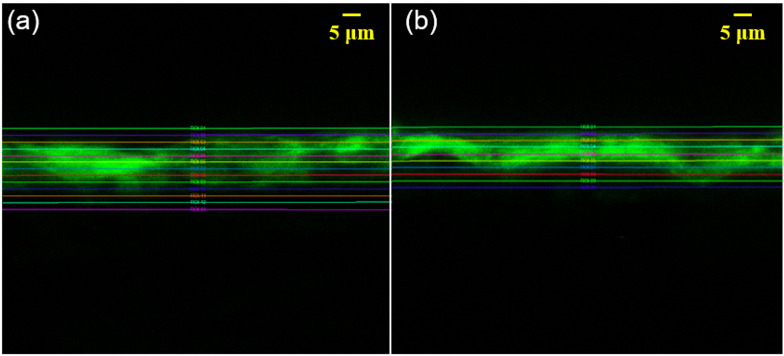
CLSM images of AO-stained PVA-coated papers: coatings on (**a**) the unsized base paper and (**b**) surface-sized base paper.

AO, a green fluorescent dye, has been used to stain fibers [14], PVA [15] and starch [16]. Hence, to investigate the penetration of PVA into the base papers, the PVA was stained with AO before the coating process. The cross-sections of the coated papers were then analyzed under the CLSM, and the intensity and distribution of the emitted fluorescence were determined and compared between the coated papers (Figure 2).

When AO-stained PVA was coated on the surface of the unsized base paper, the overall intensity of the fluorescence (Figure 2a) was not as strong as that on the surface-sized base paper (Figure 2b). Additionally, Figure 2a shows a deeper penetration of the coating into the unsized base paper, which agreed well with the results presented in Figure 1. When surface-sized paper was used as the substrate for coating, less penetration by the coating material was observed. When the same amount of coating dispersion was applied on the surface of paper, more penetration by the coating meant that less of the coating remained on the surface. This would impede film formation on the surface, and this unfavorably affects the barrier performance of polymer-coated papers.

**Figure 3:**
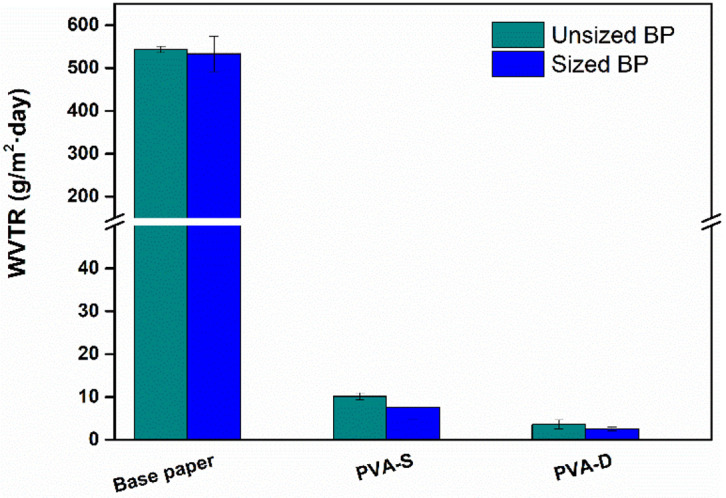
WVTR of papers. S: single coating; D: double coating; BP: base paper.

The WVTR values of the PVA-coated papers were determined to confirm the above inferences. Figure 3 shows that a PVA coating of either the unsized or surface-sized base paper reduced the WVTR substantially, relative to the base paper before PVA coating, indicating that the PVA coating is highly effective in improving the barrier performance of paper. As expected, the WVTR of the coated paper was even lower when the surface-sized base paper was used as the coating substrate, which could be attributed to the weaker penetration by coating materials and improved PVA film formation on the paper surface. Less penetration of the PVA coating into the base paper means not only an improvement in barrier performance but also a reduction in the cost of the PVA required.

### 3.3. Analysis of the Surface Coverage and Chemical Interactions

**Figure 4:**
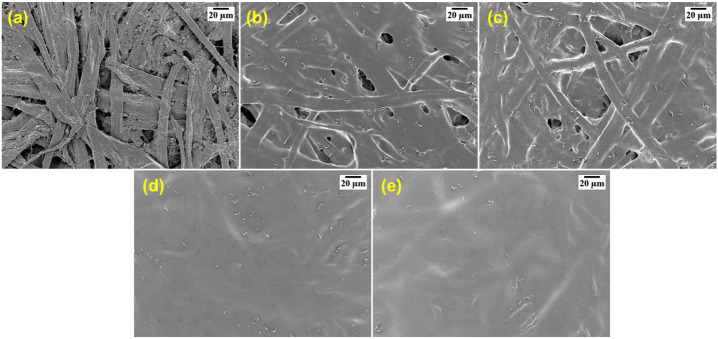
FE-SEM images of (**a**) the base paper, (**b**) PVA/AKD-S coated paper, (**c**) PVA/AKD/5% nanoclay-S coated paper, (**d**) PVA/AKD-D coated paper, and (**e**) PVA/AKD/5% nanoclay-D coated paper. S: single coating; D: double coating.

The surface-sized base paper was used in the following experiments, considering its advantage in preventing the penetration of the coating material. The surface morphologies of the base paper, PVA/AKD-coated papers and PVA/AKD/nanoclay-coated papers are shown in Figure 4, and the coat weights are presented in Table 3. The porous nature of the base paper surface, shown in Figure 4a, would allow easy penetration of fluid molecules through the typical porous structure of paper. Therefore, blocking the pores on the surface of the base paper is a simple and effective way to improve the barrier properties. A single coating of PVA/AKD (Figure 4b) failed to fully cover the surface of the base paper, and some pores remained on the surface. However, with the application of a double coating of PVA/AKD, the coating coverage of the paper surface improved and a complete sealing of all pores by the PVA/AKD film was achieved (Figure 4d). This indicates that a double coating of PVA/AKD would greatly improve the barrier performance as a well-formed film on the paper surface would prevent liquid/gas permeation. The same phenomenon was observed for the PVA/AKD/nanoclay-coated papers (Figure 4c,e), i.e., a single coating failed to seal all pores on the surface of the base paper while a double coating fully covered the surface pores and gave a satisfactory coating coverage. Considering that a continuous solid film on the surface of the paper was the key to improving barrier performance, the properties of the double-coated papers were investigated in the followed sections.

**Figure 5:**
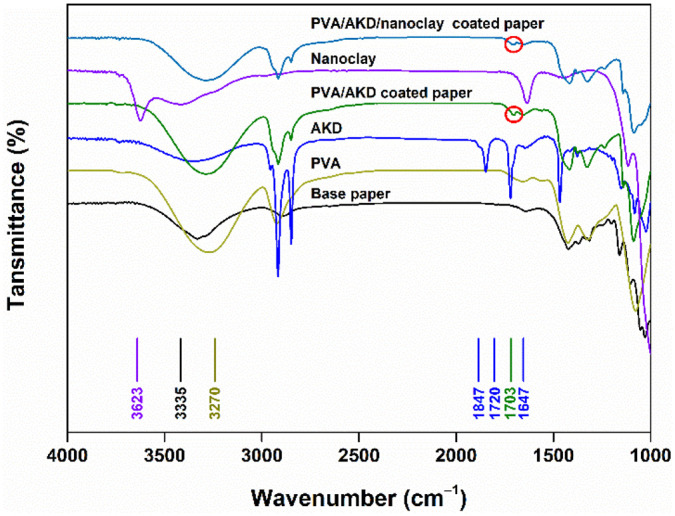
FT-IR spectra of coated papers and the coating components.

The FT-IR spectra of the coated papers are shown in Figure 5. The broad band centered at 3335 cm^−1^ in the spectrum of the base paper is attributed to the stretching vibration of −OH (hydroxyl) groups [17], and these hydroxyl groups of cellulose contribute to the hygroscopic nature of paper-based materials. Similarly, the spectrum of PVA has a strong band at 3270 cm^−1^, which can be assigned to the O−H stretching of the abundant hydroxyl groups in PVA films [18]. Therefore, although the formation of PVA film on the surface of paper can block the pores and improve the barrier performance, it also increases the hydrophilicity of paper because of the abundant hydroxyl groups of PVA. In the spectrum of AKD, the band at 1847 cm^−1^ represents the typical lactone ring of AKD molecules [19], and the bands at 1720 and 1647 cm^−1^ represent C=O and C=C stretching vibrations of AKD, respectively. A new band appeared at 1703 cm^−1^ in the spectrum of the PVA/AKD-coated paper, which indicated the formation of a β-ketoester [20]. During the drying process, AKD reacted with the hydroxyl groups of the PVA, which would enhance the hydrophobicity of the PVA-coated paper.

The spectrum of nanoclay has a strong band at 3623 cm^−1^, which can be assigned to the O–H stretching of Al−OH and Si−OH structures of nanoclay [21]. Interestingly, this band was not visible in the spectrum of the PVA/AKD/nanoclay-coated paper, possibly because nanoclay was embedded in the PVA/AKD matrix and could not be detected. Meanwhile, the band at 1703 cm^−1^ (β-ketoester) was also visible in the spectrum of the PVA/AKD/nanoclay-coated paper. This again suggests that chemical reactions occurred between AKD and the hydroxyl groups of PVA, which may have resulted in the improved water resistance of the paper.

### 3.4. Water Resistance and Barrier Performance of Coated Papers

**Figure 6:**
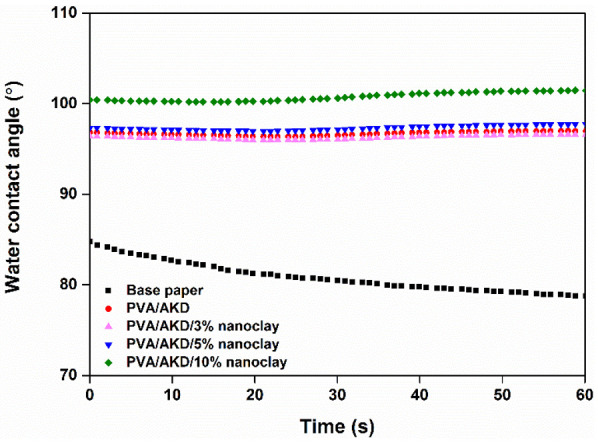
WCAs of the base paper and coated papers.

The WCAs of the coated papers were determined and compared with the WCA of the base paper (Figure 6). Although the base paper was surface-sized, the WCA slightly decreased with time. When the AKD was incorporated into the coating formulation, the WCAs of the coated papers increased, showing the effectiveness of AKD in increasing the water repellency of paper. The hydrophobizing agent AKD can react with the hydroxyl group of PVA and cellulose to form ester bonds [22], and thus decrease the hydrophilicity of the coated paper, which was confirmed in the FT-IR analysis (Figure 5). The addition of nanoclay slightly increased the WCA of the coated papers, and it appeared that the change in surface morphology of the coated papers contributed to this phenomenon [23]. Importantly, the WCAs of the coated papers remained constant during the testing, indicating good water resistance was achieved, which would enhance the applicability of the resulting coated papers in humid or even wet conditions.

**Figure 7:**
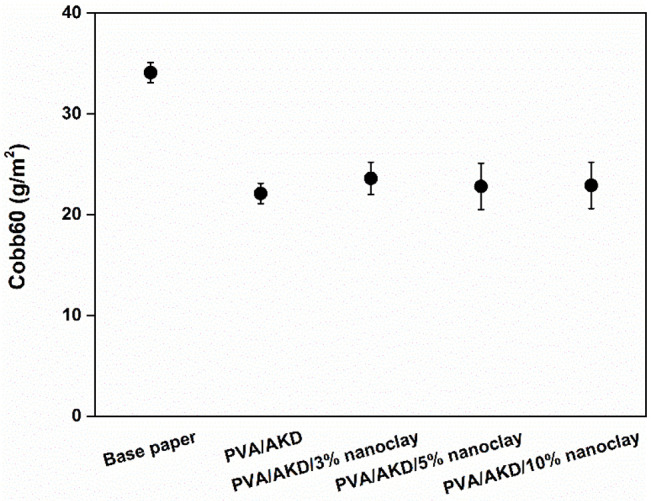
Cobb60 values of the base paper and coated papers.

A Cobb value represents the amount of water absorbed by paper during a specific period, such as 60 s in this study. The value is affected by various characteristics of the paper, such as the porosity and sizing. A smaller Cobb value indicates better water resistance of the coated paper. Figure 7 shows that the Cobb60 values of the coated papers were much lower than that of the base paper because the PVA coating layers decreased both the number and the size of the pores. Additionally, Hu et al. [24] showed that eliminating the pores in paper was an effective method of improving water resistance. This was consistent with the present study’s finding that sizing remarkably reduced the water absorption of paper, as shown in Figure 1b. These results give insight into controlling water resistance by altering the characteristics of paper.

**Figure 8:**
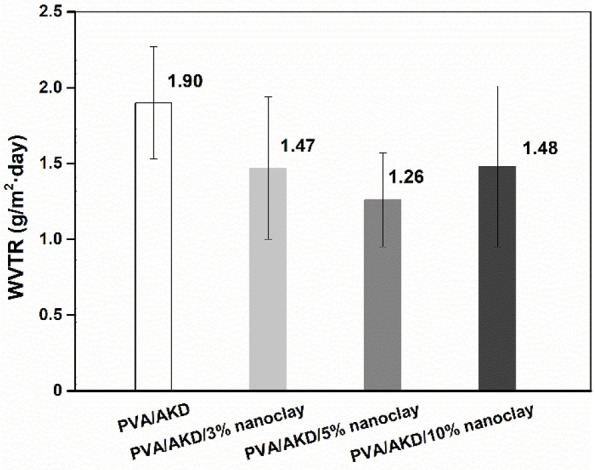
WVTR of the base paper and coated papers.

The WVTR is an important barrier property of packaging materials for moisture-sensitive products. The WVTRs of the base paper and coated papers were examined (Figure 8). Compared with the base paper, the coated papers had much lower WVTRs. PVA formed a dense polymer film on the paper surface and decreased the number of pores. A higher coat weight further improved the barrier performance of the papers (Figure 3) because it provided more complete coverage of the paper surface. There was no big difference in the absolute values of WVTR for the coated papers because the coating of PVA/AKD had already decreased the WVTR of paper to a low level (1.90 g/m^2^·day). The use of 3% nanoclay in the coating formulation further decreased the WVTR value to 1.47 g/m^2^·day, which was 22.6% lower than that of PVA/AKD coated paper. The use of 5% nanoclay decreased the WVTR value to 1.26 g/m^2^·day, which was 33.7% lower than that of PVA/AKD coated paper. These improvements can be attributed to the tortuosity effect of this platy nano-sized material [25], i.e., the presence of nanoclay in a PVA-based coating prevents the diffusion of permeating molecules by making the molecules follow a more tortuous pathway. However, when 10% nanoclay was used, the WVTR of the coated paper increased. It is known that the tortuosity effect is dependent on the dispersion of the nanoclay in the coating materials. When an excessive amount of nanoclay is present in the polymer matrix, an agglomeration of nanoclay generates interfacial voids at the particle/polymer matrix interface and forms a preferential pathway for the diffusion of water vapor molecules [26]. In the present study, the addition of 5% nanoclay gave the optimal water vapor resistance, suggesting that neither insufficient nor excess use of nanoclay provides an ideal tortuosity effect owing to the lack of nanoplates or agglomeration of nanoclay in the diffusion pathway of water vapor [27]. Thus, selecting suitable nanofillers and optimizing their dosage is critical for obtaining a coated paper with high barrier performance.

In our previous work [9], the barrier properties of papers were enhanced by applying different layers of PVA/AKD coatings on the surface of a base paper. The lowest WVTR value was around 2 g/m^2^·day when the triple coating of PVA/AKD (11 g/m^2^ coat weight) was applied on the surface of the base paper. However, it was difficult to further decrease the WVTR by coating more PVA/AKD on the surface of the triple-coated paper, i.e., quadruple coating (15 g/m^2^ coat weight). Interestingly, a lower WVTR value was achieved by double coating (12 g/m^2^ coat weight) of PVA/AKD/5% nanoclay on the surface of the paper, which again confirmed the effectiveness of using nanoclay. 

**Figure 9:**
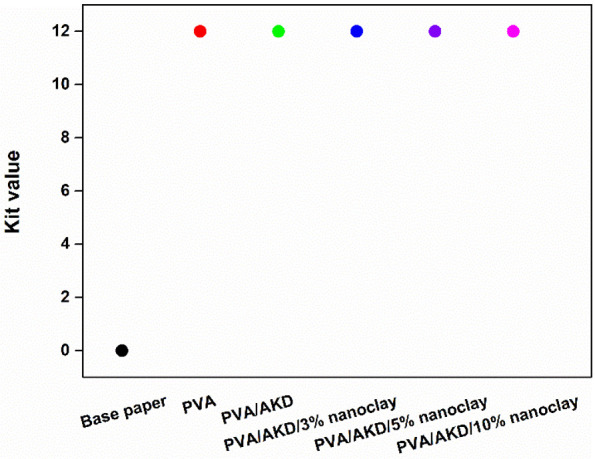
Kit values of the base paper and coated papers in grease resistance testing.

Grease resistance is another major factor affecting the barrier performance of packaging papers, especially for the packaging of oily foods and certain machinery accessories. The grease resistance of the papers was determined according to TAPPI standard T 559 cm-12 (Figure 9), where a kit value ranging from 0 to 12 is used to grade the repellency of paper to grease and oil. A higher kit value indicates a greater grease resistance of a paper. The results showed the base paper had a kit value of 0, indicating that it has no grease resistance. In contrast, all coated papers had a kit value of 12, showing an extraordinary improvement in grease resistance. Two factors contributed to the satisfactory grease resistance of these coated papers. First, PVA itself has strong grease resistance [28]. This is seen from the kit value of the PVA-coated paper (red dot in Figure 9). Second, the sealing of the pores on the surface of base paper prevents the penetration of grease [29]. A PVA coating improves the resistance against grease by forming a continuous film on paper surfaces with high oil resistance. One thing to point out is that the same kit values were always obtained in the grease resistance test of each sample. For example, in the grease resistance test of the base paper, a kit value of zero was obtained in each replicate measurement, suggesting the poor grease resistance of the sample and the good repeatability of the experiment. Therefore, the error bar is not visible in Figure 9.

### 3.5. Strength Properties of Coated Papers

**Figure 10:**
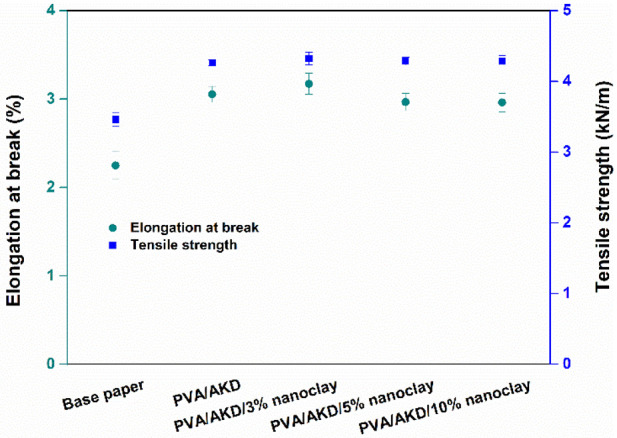
Elongations at break and tensile strengths of the base paper and coated papers.

The physical properties of papers play an important role in the converting process and end uses of the papers. For example, the tensile strength and extensibility of papers are indicative of the potential resistance to the web break of papers in a printing process or any other web-fed converting process [30]. In the case of sack paper, tensile strength and elongation affect how strong this packaging material can be and how much load it can carry. The elongation at break and tensile strength of the papers examined in this study are depicted in Figure 10. The base paper had an elongation at break of 2.2% and tensile strength of 3.5 kN/m, which were much lower than the values for the coated papers. Specifically, the elongation at break of all coated papers was at least 32% higher than that of the base paper, and the tensile strength of coated papers was 23–25% higher than that of the base paper. This indicates that the coated paper can withstand a stronger stretching or deformation without breaking compared to the base paper. The tensile strength improved after PVA-based coating because PVA has a good reinforcing effect on the tensile strength of paper [31,32]. When nanoclay was used in the coating formulation, the mechanical properties of the coated papers remained similar. Thus, a conclusion can be made that the improvement in the elongation and tensile strength was mainly because of the PVA/AKD films. In other words, the use of nanoclay had little influence on the mechanical properties of coated papers. Our previous work showed that the coating of PVA/AKD improved water and grease resistance, and the mechanical properties [9].

## 4. Conclusions

In this work, the barrier performance and water resistance of cellulosic paper were enhanced by coating a PVA/AKD/nanoclay dispersion on the surface of paper. The effect of the absorptive characteristics of the base papers on the improvement in barrier performance was investigated by CLSM analysis. The results indicated that the use of surface-sized base paper contributed to a higher barrier performance owing to the impeded penetration of coatings and thus a favored film formation on the paper surface. The use of AKD in the coating formulation improved the water resistance of the papers, and the coated papers had WCAs of around 100°. The addition of 5% nanoclay in the coating formulation further enhanced the barrier performance of cellulosic paper owing to its tortuosity effect on the diffusion pathway, and the lowest WVTR value of a coated paper was 1.3 g/m^2^·day when a double coating of PVA/AKD/5% nanoclay dispersion was applied. The water and grease resistance of the coated papers improved notably, relative to those of the base paper. In summary, the PVA/AKD/nanoclay-coated papers exhibited hydrophobicity, excellent barrier performance and improved strength properties, which would expand the applications of cellulosic paper in the packaging industry.

## Figures and Tables

**Table 1:** Comparison of the properties of the two base papers.

Base Paper	Surface-Unsized	Surface-Sized
Basis weight (g/m^2^) Thickness (μm)	84	81
	99.25	97.12
Bulk (cm^3^/g)	1.18	1.20
Density (g/cm^3^)	0.85	0.83
PPS ^1^ roughness (μm)	4.53	5.46
Brightness (%)	92.28	92.81
WVTR ^2^ (g/m^2^∙day)	543.11	533.01

^1^ PPS = Parker Print-Surf, ^2^ WVTR = water vapor transmission rate.

**Table 2:** Coating formulations.

	Coating	#1	#2	#3	#4
Component					
PVA (pph ^1^)		100	100	100	100
AKD (pph)		15	15	15	15
Nanoclay (pph)		0	3	5	10

^1^ Component amounts are reported as parts per hundred (pph; by weight) based on 100 parts of PVA. PVA = polyvinyl alcohol; AKD = alkyl ketene dimer.

**Table 3:** Coat weights of the papers.

	Coated Paper	Single Coating	Double Coating
Formulation			
PVA/AKD		6.51 ± 0.17 ^1^	11.79 ± 0.16
PVA/AKD/3% nanoclay		6.55 ± 0.08	12.28 ± 0.17
PVA/AKD/5% nanoclay		6.52 ± 0.16	12.08 ± 0.16
PVA/AKD/10% nanoclay		6.62 ± 0.16	12.46 ± 0.21

^1^ The coat weight is reported as g/m^2^.

## Data Availability

The data presented in this study are available on request from the corresponding author.
